# No association of ABO blood groups and Rh factor with primary liver cancer in cirrhotic patients: a single-center cross-sectional study

**DOI:** 10.3389/fmed.2024.1432137

**Published:** 2025-01-22

**Authors:** Liyan Dong, Yuhang Yin, Huiyuan Lu, Di Sun, Dongyang Wang, Deli Zou, Xingshun Qi

**Affiliations:** ^1^Department of Gastroenterology, General Hospital of Northern Theater Command, Shenyang, China; ^2^Postgraduate College, China Medical University, Shenyang, China; ^3^Postgraduate College, Shenyang Pharmaceutical University, Shenyang, China; ^4^Postgraduate College, Dalian Medical University, Dalian, China

**Keywords:** ABO blood groups, rhesus factor, primary liver cancer, liver cirrhosis, risk factor

## Abstract

**Background:**

Primary liver cancer (PLC) is one of the most common cancers worldwide. ABO blood groups and rhesus (Rh) factor are inherited characteristics. Their association with the presence of PLC remains unclear in cirrhotic patients. Hence, the purpose of this cross-sectional study was to evaluate whether blood groups were risk factors for the presence of PLC in cirrhosis.

**Methods:**

Patients with liver cirrhosis who were consecutively admitted to the Department of Gastroenterology of the General Hospital of Northern Theater Command from 1 January 2010 to 30 June 2014 were retrospectively screened. Logistic regression analyses were performed to explore the association of ABO blood groups and Rh factor with PLC in cirrhotic patients. Adjusted odds ratios (aORs) with 95% confidence intervals (CIs) were calculated after adjusting for gender, age, family history of liver cirrhosis, HBV-DNA positivity, and etiology of cirrhosis. Subgroup analyses were performed according to the etiology of liver cirrhosis.

**Results:**

Overall, 1,158 cirrhotic patients without PLC and 240 cirrhotic patients with PLC were included in the study. After adjusting for confounding factors, non-O (aOR = 0.763; 95%CI = 0.449–1.298, *p* = 0.319), A (aOR = 0.643; 95%CI = 0.332–1.246, *p* = 0.191), B (aOR = 0.835; 95%CI = 0.453–1.540, *p* = 0.564), AB (aOR = 0.888; 95%CI = 0.363–2.170, *p* = 0.795), and Rh (+) (aOR = 0.239; 95%CI = 0.036–1.571, *p* = 0.136) blood groups were not independently associated with PLC in cirrhotic patients. In the subgroup analysis of HBV-related cirrhotic patients, the proportion of A blood group was significantly lower in cirrhotic patients with PLC than in those without PLC (24.17% vs. 33.99%, *p* < 0.001); however, in HCV- and alcohol-related cirrhotic patients, the proportions of ABO blood groups and Rh factor were not significantly different between the two groups.

**Conclusion:**

ABO blood groups and Rh factor may not be associated with the presence of PLC in cirrhotic patients.

## 1 Introduction

Primary liver cancer (PLC) is the sixth most common cancer and the third leading cause of cancer-related death worldwide in 2020, with approximately 906,000 new cases and 830,000 deaths (1). PLC primarily occurs as a consequence of chronic liver diseases, including hepatitis B virus (HBV) or hepatitis C virus (HCV) infection and alcoholic or non-alcoholic fatty liver diseases (2). Smoking, obesity, diabetes, iron overload, and aflatoxin B1 exposures are also identified as major risk factors associated with PLC (3). Except for environmental and lifestyle-related factors, epigenetics also plays an important role in the pathogenesis of PLC (4). The ABO blood groups and rhesus (Rh) factor are inherited characteristics, and the expression of ABO and Rh antigens varies among individuals. Nowadays, several malignancies, including pancreatic (5), gastric (6), skin (7), and ovarian (8) cancers, are assumed to be associated with ABO blood groups and Rh factor. In recent years, there is a growing body of evidence on the association of ABO blood groups and Rh factor with PLC (9–12), but their relationship remains controversial. Li et al. found that A blood group was associated with a higher risk of HCV-related hepatocellular carcinoma (HCC) (9). Similarly, Li et al. found that after adjusting for age, sex, type 2 diabetes, cirrhosis, hepatitis B e antigen, and HBV-DNA, A blood group was associated with a higher risk of HBV-related HCC (10). However, Lu et al. found that ABO blood groups were not associated with HBV-related HCC (11). Huang et al. found that AB blood group was associated with a higher risk of HCC (12).

To date, the association of ABO blood groups and Rh factor with the risk of developing PLC in cirrhotic patients is still unclear. To the best of our knowledge, only one study by Iavarone et al. which retrospectively included 215 cirrhotic patients without HCC and 194 cirrhotic patients with HCC, found that non-O blood group was significantly associated with a higher risk of HCC. However, this association was weak probably due to a limited sample size (13). In this setting, we carried out a cross-sectional study to evaluate the association of ABO blood groups and Rh factor with the presence of PLC in cirrhotic patients.

## 2 Methods

### 2.1 Study design

We retrospectively reviewed the medical records of patients with liver cirrhosis who were consecutively admitted to the Department of Gastroenterology of the General Hospital of Northern Theater Command from 1 January 2010 to 30 June 2014. This study was carried out following the rules of the 1975 Declaration of Helsinki and approved by the Medical Ethical Committee of the General Hospital of Northern Theater Command with an approval number [Y (2024) 008]. Patients’ written informed consents were waived by the Medical Ethical Committee of the General Hospital of Northern Theater Command due to the retrospective nature of this study.

The exclusion criteria were as follows: (i) repeated admissions of the same patient; (ii) age < 18 years; (iii) patients who did not have sufficient data about ABO blood groups; (iv) patients who had a history or evidence of other non-hepatic malignancy; and (v) patients who did not have imaging-based evidence for a definite diagnosis of liver cirrhosis during their hospitalizations.

### 2.2 Diagnosis and definitions

Liver cirrhosis was diagnosed based on clinical manifestations, laboratory tests, imaging, liver stiffness measurement, and histopathological examinations, if necessary. PLC was primarily diagnosed by findings from contrast-enhanced computed tomography (CT) and/or magnetic resonance imaging (MRI) scans with or without a serum alpha-fetoprotein (AFP) level of greater than 400 ng/mL, or histology, if necessary (2, 10).

According to the presence of A and B antigens on the red blood cells (RBC), the ABO blood group includes (i) A blood group characterized as anti-A (+) and anti-B (−); (ii) B blood group as anti-A (−) and anti-B (+); (iii) O blood group as anti-A (−) and anti-B (−); and (iv) AB blood group as anti-A (+) and anti-B (+). According to the presence or absence of D antigen on the RBC, the Rh factor includes (i) Rh (+) blood group characterized as anti-D (+) and (ii) Rh (−) blood group as anti-D (−).

### 2.3 Data collection

Demographic, clinical, and laboratory data at admissions were collected, including age, gender, smoking, drinking, etiology of cirrhosis (i.e., HBV infection, HCV infection, and alcohol abuse), hypertension, diabetes, family history of liver cirrhosis, ABO blood groups, Rh factor, red blood cells (RBC), hemoglobin (Hb), white blood cell (WBC), platelet count (PLT), total bilirubin (TBIL), albumin (ALB), alanine aminotransferase (ALT), aspartate aminotransferase (AST), alkaline phosphatase (AKP), gamma-glutamyltransferase (GGT), serum creatinine (Scr), blood urea nitrogen (BUN), serum sodium (Na), international normalized ratio (INR), activated partial thromboplastin time (APTT), and AFP. Child–Pugh score and class and Model for End-Stage Liver Disease (MELD) score were also calculated.

### 2.4 Statistical analyses

All statistical analyses were performed using SPSS version 26.0 statistical software (IBM Corp, Armonk, New York, USA). Continuous variables were expressed as mean ± standard deviation and median (range) and compared using the independent sample *t*-tests for normal distribution or the Mann–Whitney *U*-tests for non-normal distribution. Categorical variables were expressed as frequency (percentage), and their difference between the groups was evaluated using Chi-squared or Fisher’s exact tests. Logistic regression analyses were used to explore whether ABO blood groups and Rh factor were significantly associated with PLC in cirrhotic patients. Crude odds ratios (cORs) with 95% confidence intervals (CIs) were calculated in univariate analyses. Adjusted odds ratios (aORs) with 95%CIs were calculated after adjusting for gender, age, family history of liver cirrhosis, HBV-DNA, and etiology of cirrhosis. Finally, subgroup analyses were performed to explore the association of ABO blood groups and Rh factor with PLC in patients with different etiologies of liver cirrhosis. Interactions between etiologies of liver cirrhosis and ABO blood groups or Rh factor were tested in subgroup analyses, if appropriate. A two-tailed *p* < 0.05 was considered statistically significant.

## 3 Results

### 3.1 Description of overall patients

**Figure 1:**
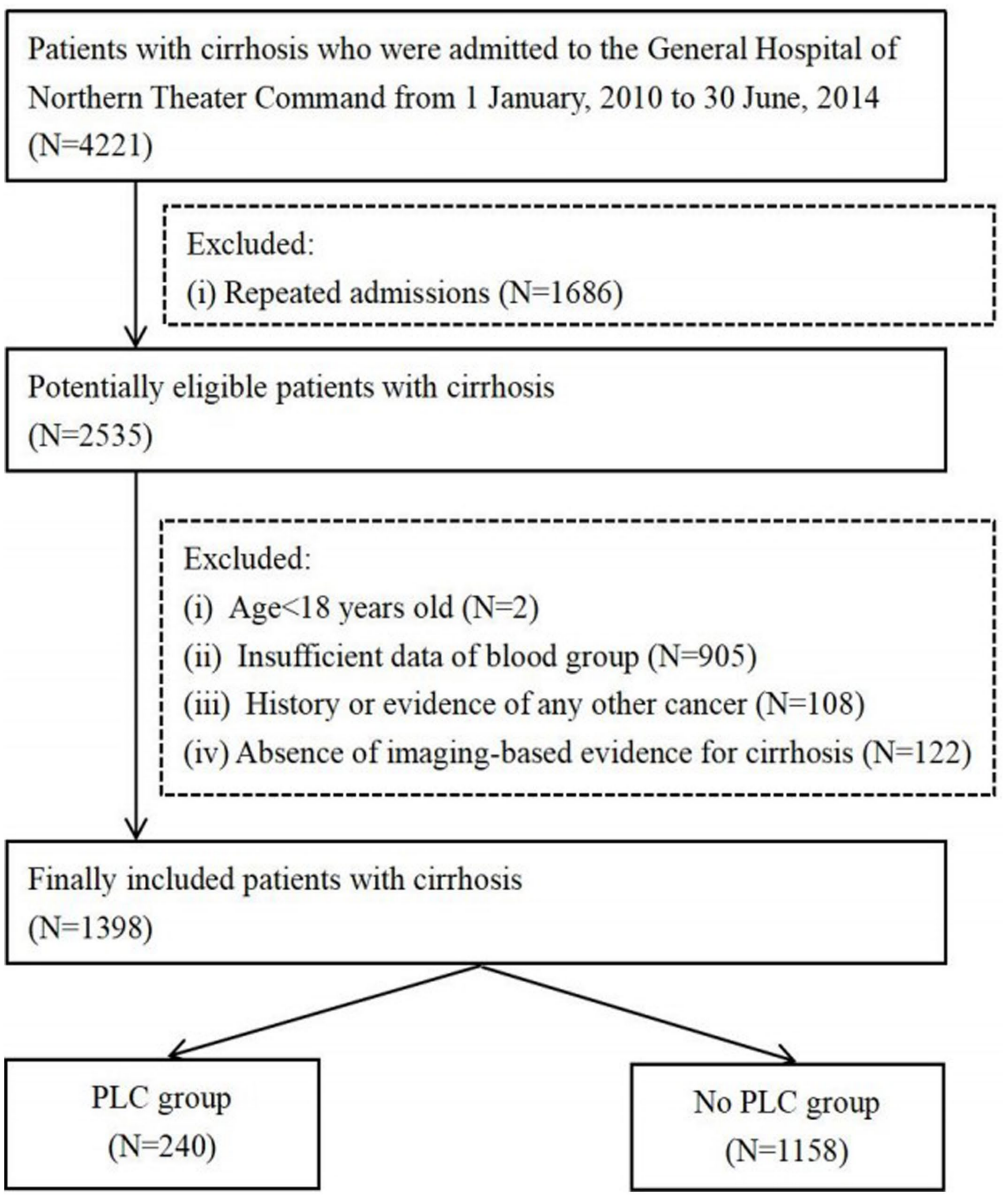
Flowchart of patients selection.

A total of 1,158 cirrhotic patients without PLC and 240 cirrhotic patients with PLC were included in the study (Figure 1). The mean age of cirrhotic patients without and with PLC was 56.08 and 58.56 years, respectively, and the proportion of male subjects without and with PLC was 66.84% (774/1158) and 79.17% (190/240), respectively.

### 3.2 Overall comparison between cirrhotic patients without and with PLC

The proportions of male subjects (66.84% vs. 79.17%, *p* < 0.001) and HBV-DNA ≥ 1 × 10^3^ (22.97% vs. 37.70%, *p* = 0.001), age (56.08 ± 11.82 years vs. 58.56 ± 11.06 years, *p* = 0.005), and AFP level (8.91 ± 39.26 vs. 78.51 ± 138.88, *p* < 0.001) were significantly different between cirrhotic patients without and with PLC. Cirrhotic patients with PLC had a significantly higher prevalence of HBV infection alone (50.00% vs. 30.74%, *p* < 0.001) but a lower prevalence of alcohol abuse alone (12.08% vs. 23.40%, *p* < 0.001) than those without. The proportions of A (28.41% vs. 25.00%, *p* = 0.283), B (32.56% vs. 33.33%, *p* = 0.815), O (28.15% vs. 32.08%, *p* = 0.221), AB (10.88% vs. 9.58%, *p* = 0.553), and Rh (+) (99.30% vs. 98.60%, *p* = 0.569) blood groups were not significantly different between cirrhotic patients without and with PLC (Table 1).

**Table 1:** Comparison between cirrhotic patients without and with PLC.

Variables	Cirrhosis without PLC		Cirrhosis with PLC		*p*-value
	No. Pts	Median (range), Mean ± SD or Frequency (percentage)	No. Pts	Median (range), Mean ± SD or Frequency (percentage)	
**Demographics**					
Age (years)	1,158	55.66 (21.19–95.13); 56.08 ± 11.82	240	57.16 (23.80–88.29); 58.56 ± 11.06	**0.005**
Male	1,158	774 (66.84%)	240	190 (79.17%)	**<0.001**
**Smoking (%)**	850	276 (32.47%)	191	71 (37.17%)	0.213
**Drinking (%)**	856	345 (40.30%)	192	77 (40.10%)	0.959
**Family history of liver cirrhosis (%)**	1,158	16 (1.40%)	240	8 (3.33%)	0.065
**Hypertension (%)**	1,158	160 (13.82%)	240	36 (15.00%)	0.631
**Diabetes (%)**	1,158	212 (18.31%)	240	43 (17.92%)	0.887
**Etiology of liver cirrhosis**					
HBV infection alone (%)	1,158	356 (30.74%)	240	120 (50.00%)	**<0.001**
HCV infection alone (%)	1,158	79 (6.82%)	240	23 (9.58%)	0.134
Alcohol abuse alone (%)	1,158	271 (23.40%)	240	29 (12.08%)	**<0.001**
**ABO blood groups (%)**					
O (%)	1,158	326 (28.15%)	240	77 (32.08%)	0.221
A (%)	1,158	329 (28.41%)	240	60 (25.00%)	0.283
B (%)	1,158	377 (32.56%)	240	80 (33.33%)	0.815
AB (%)	1,158	126 (10.88%)	240	23 (9.58%)	0.553
**Rh (+) (%)**	1,091	1,083 (99.30%)	214	211 (98.60%)	0.569
**Laboratory parameters**					
HBV-DNA ≥1 × 10^3^ (%)	518	119 (22.97%)	122	46 (37.70%)	**0.001**
RBC (10^12^/L)	1,151	3.04 (0.93–6.78); 3.08 ± 0.87	235	3.60 (1.28–5.75); 3.52 ± 0.87	**<0.001**
Hb (g/L)	1,151	89.00 (27.00–218.00); 92.43 ± 30.22	235	113.00 (31.00–169.00); 110.49 ± 28.05	**<0.001**
WBC (10^9^/L)	1,151	4.20 (0.30–46.10); 5.35 ± 4.20	235	5.00 (1.10–38.00); 6.09 ± 4.08	**<0.001**
PLT (10^9^/L)	1,151	74.00 (5.00–592.00); 94.34 ± 71.45	235	90.00 (23.00–437.00); 109.49 ± 66.96	**<0.001**
TBIL (μmol/L)	1,146	21.80 (2.00–809.80); 43.03 ± 71.11	234	23.55 (6.30–491.90); 47.99 ± 72.16	0.315
ALB (g/L)	1,125	31.90 (0.40–52.80); 31.91 ± 7.18	235	34.00 (14.00–48.90); 33.29 ± 6.90	**0.007**
AST (U/L)	1,145	36.00 (8.00–1487.00); 60.20 ± 113.38	234	57.00 (14.00–663.00); 85.33 ± 90.49	**<0.001**
ALT (U/L)	1,145	26.00 (3.00–1335.00); 40.96 ± 73.05	234	39.00 (9.00–762.00); 57.91 ± 71.60	**<0.001**
AKP (U/L)	1,143	82.00 (7.05–969.00); 107.94 ± 88.10	234	111.00 (30.00–769.00); 142.58 ± 107.40	**<0.001**
GGT (U/L)	1,143	44.00 (5.00–4562.00); 105.91 ± 209.76	234	100.00 (8.00–1091.00); 146.39 ± 148.37	**<0.001**
Scr (umol/L)	1,130	60.95 (20.00–1069.00); 82.02 ± 98.06	230	60.30 (25.00–355.00); 72.22 ± 42.05	0.559
BUN (mmol/L)	1,129	5.92 (1.63–61.88); 7.98 ± 6.88	230	5.75 (2.06–41.58); 7.16 ± 4.62	0.371
Na (mmol/L)	1,144	139.00 (116.40–160.80); 138.43 ± 4.70	233	138.60 (112.10–147.90); 137.72 ± 5.08	0.107
INR	1,127	1.26 (0.77–5.94); 1.39 ± 0.53	232	1.16 (0.84–4.33); 1.28 ± 0.46	**<0.001**
APTT (s)	1,125	42.00 (21.90–168.00); 43.47 ± 9.79	233	41.60 (28.20–105.30); 42.37 ± 8.79	0.064
AFP (IU/ml)	751	2.96 (0.28–802.60); 8.91 ± 39.26	138	9.79 (0.23–606.90); 78.51 ± 138.88	**<0.001**
**Child–Pugh score**	1,090	7.00 (5.00–15.00); 7.68 ± 2.21	224	7.00 (5.00–14.00); 7.35 ± 2.20	**0.028**
**Child–Pugh class**					
A (%)	1,090	397 (36.42%)	224	96 (42.86%)	0.070
B (%)	1,090	465 (42.66%)	224	88 (39.29%)	0.351
C (%)	1,090	228 (20.92%)	224	40 (17.86%)	0.301
**MELD score**	1,105	9.54 (6.43–40.00); 12.41 ± 7.57	225	8.19 (6.43–31.38); 10.05 ± 5.00	**<0.001**

No. Pts, number of patients; PLC, primary liver cancer; SD, standard deviation; HBV, hepatitis B virus; HCV, hepatitis C virus; RBC, red blood cell; Hb, hemoglobin; WBC, white blood cell; PLT, platelet count; TBIL, total bilirubin; ALB, albumin; AST, aspartate aminotransferase; ALT, alanine aminotransferase; AKP, alkaline phosphatase; GGT, gamma-glutamyltransferase; Scr, serum creatinine; BUN, blood urea nitrogen; Na, serum sodium; INR, international normalized ratio; APTT, activated partial thromboplastin time; AFP, alpha-fetoprotein; MELD, Model of End-Stage Liver Disease. Notes to bold values: There were significant differences between the two groups.

Collectively, ABO blood groups and Rh factor were not associated with PLC in overall patients.

### 3.3 Logistic regression analyses regarding the association of ABO blood groups and Rh factor with PLC in liver cirrhosis

Univariate logistic regression analyses showed that male subjects (cOR = 1.885, 95%CI = 1.349–2.635, *p* < 0.001), age ≥ 60 years (cOR = 1.452, 95%CI = 1.094–1.927, *p* = 0.010), family history of liver cirrhosis (cOR = 2.461, 95%CI = 1.041–5.818, *p* = 0.040), HBV-DNA ≥ 1 × 10^3^ (cOR = 2.029, 95%CI = 1.334–3.087, *p* = 0.001), HBV infection alone (cOR = 3.150, 95%CI = 2.038–4.868, p < 0.001), and HCV infection alone (cOR = 2.721, 95%CI = 1.490–4.967, p = 0.001) were significantly associated with PLC in cirrhotic patients (Table 2).

**Table 2:** Univariate logistic regression analyses for risk factors of PLC.

Variables	Univariate analysis	
	Crude OR (95% CI)	*p*-value
**Sex**		**<0.001**
Female	1	
Male	1.885 (1.349–2.635)	
**Age**		**0.010**
<60	1	
≥60	1.452 (1.094–1.927)	
**Family history of liver cirrhosis**		**0.040**
No	1	
Yes	2.461 (1.041–5.818)	
**History of smoking**		0.213
No	1	
Yes	1.230 (0.888–1.706)	
**History of drinking**		0.959
No	1	
Yes	0.992 (0.721–1.365)	
**Diabetes**		0.887
No	1	
Yes	0.974 (0.678–1.399)	
**Hypertension**		0.631
No	1	
Yes	1.101 (0.744–1.628)	
**HBV-DNA**		**0.001**
<1 × 10^3^	1	
≥1 × 10^3^	2.029 (1.334–3.087)	
**Etiology of liver cirrhosis**		
Alcohol abuse alone (%)	1	
HBV infection alone (%)	3.150 (2.038–4.868)	**<0.001**
HCV infection alone (%)	2.721 (1.490–4.967)	**0.001**
**Child–Pugh class**		
A (%)	1	
B (%)	0.783 (0.569–1.076)	0.132
C (%)	0.726 (0.485–1.086)	0.119
**Laboratory parameters**		
RBC (10^12^/L)	1.791 (1.519–2.113)	**<0.001**
Hb (g/L)	1.020 (1.015–1.025)	**<0.001**
WBC (10^9^/L)	1.038 (1.007–1.069)	**0.015**
PLT (10^9^/L)	1.003 (1.001–1.004)	**0.003**
TBIL (μmol/L)	1.001 (0.999–1.003)	0.334
ALB (g/L)	1.028 (1.007–1.049)	**0.007**
ALT (U/L)	1.002 (1.001–1.004)	**0.005**
AST (U/L)	1.002 (1.000–1.003)	**0.004**
Scr (umol/L)	0.998 (0.996–1.001)	0.150
BUN (mmol/L)	0.977 (0.952–1.003)	0.085
Na (mmol/L)	0.970 (0.943–0.999)	**0.040**
INR	0.553 (0.376–0.815)	**0.003**
APTT (s)	0.986 (0.970–1.003)	0.111
**Child–Pugh score**	0.933 (0.872–0.998)	**0.045**
**MELD score**	0.940 (0.913–0.967)	**<0.001**

PLC, primary liver cancer; HBV, hepatitis B virus; HCV, hepatitis C virus; RBC, red blood cell; Hb, hemoglobin; WBC, white blood cell; PLT, platelet count; TBIL, total bilirubin; ALB, albumin; ALT, alanine aminotransferase; AST, aspartate aminotransferase; Scr, serum creatinine; BUN, blood urea nitrogen; Na, serum sodium; INR, international normalized ratio; APTT, activated partial thromboplastin time; MELD, Model of End-Stage Liver Disease. Notes to bold values: There were significant differences between the two groups.

Compared with O blood group, non-O (cOR = 0.829, 95%CI = 0.615–1.119, *p* = 0.221), A (cOR = 0.772, 95%CI = 0.533–1.119, *p* = 0.171), B (cOR = 0.898, 95%CI = 0.635–1.270, *p* = 0.544), and AB (cOR = 0.773, 95%CI = 0.465–1.286, *p* = 0.321) blood groups were not significantly associated with PLC in cirrhotic patients. Multivariate logistic regression analyses also showed that non-O (aOR = 0.763, 95%CI = 0.449–1.298, *p* = 0.319), A (aOR = 0.643, 95%CI = 0.332–1.246, *p* = 0.191), B (aOR = 0.835, 95%CI = 0.453–1.540, *p* = 0.564), and AB (aOR = 0.888, 95%CI = 0.363–2.170, *p* = 0.795) blood groups were not independently associated with PLC in cirrhotic patients (Table 3).

**Table 3:** Univariate and multivariate logistic regression analyses of ABO blood groups and Rh factor in cirrhotic patients with PLC.

Variables	Univariate analysis		Multivariate analysis**	
	Crude OR (95% CI)	*p*-value	Adjusted OR (95% CI)	*p*-value
**ABO blood groups**				
O	1			
Non-O	0.829 (0.615–1.119)	0.221	0.763 (0.449–1.298)	0.319
A	0.772 (0.533–1.119)	0.171	0.643 (0.332–1.246)	0.191
B	0.898 (0.635–1.270)	0.544	0.835 (0.453–1.540)	0.564
AB	0.773 (0.465–1.286)	0.321	0.888 (0.363–2.170)	0.795
**Rh factor**				
Rh (−)	1			
Rh (+)	0.520 (0.137–1.974)	0.336	0.239 (0.036–1.571)	0.136

^**^Adjusted for gender, age, family history of liver cirrhosis, HBV-DNA, and etiology of liver cirrhosis. PLC, primary liver cancer. Notes to bold values: There were significant differences between the two groups.

Compared with Rh (−) blood group, Rh (+) blood group (cOR = 0.520, 95%CI = 0.137–1.974, *p* = 0.336) was not significantly associated with PLC in cirrhotic patients. Multivariate logistic regression analysis showed that Rh factor (aOR = 0.239, 95%CI = 0.036–1.571, *p* = 0.136) was not independently associated with PLC in cirrhotic patients (Table 3).

Collectively, ABO blood groups and Rh factor were not significantly associated with PLC in cirrhotic patients.

### 3.4 Subgroup comparison between cirrhotic patients without and with PLC

#### 3.4.1 HBV infection alone

In the subgroup of patients with HBV infection alone, the proportion of A blood group was significantly higher in cirrhotic patients without PLC than those with PLC (33.99% vs. 24.17%, *p* < 0.001), but the proportions of B, O, AB, and Rh (+) blood groups were not significantly different between cirrhotic patients without and with PLC (Table 4).

**Table 4:** Comparison of the proportions of ABO blood groups and Rh factor in subgroup analyses between cirrhotic patients without and with PLC.

Subgroups	Cirrhosis without PLC		Cirrhosis with PLC		*p*-value
	No. Pts	Frequency (percentage)	No. Pts	Frequency (percentage)	
**HBV infection alone**					
O	356	85 (23.88%)	120	38 (31.67%)	0.092
A	356	121 (33.99%)	120	29 (24.17%)	**<0.001**
B	356	110 (30.90%)	120	40 (33.33%)	0.620
AB	356	40 (11.24%)	120	13 (10.83%)	0.903
Rh (+)	337	335 (99.41%)	111	110 (99.10%)	0.575
**HCV infection alone**					
O	79	25 (31.65%)	23	8 (34.78%)	0.777
A	79	18 (22.78%)	23	7 (30.43%)	0.453
B	79	27 (34.18%)	23	5 (21.74%)	0.258
AB	79	9 (11.39%)	23	3 (13.04%)	1.000
Rh (+)	73	72 (98.63%)	21	21 (100%)	1.000
**Alcohol abuse alone**					
O	271	83 (30.63%)	29	9 (31.03%)	0.964
A	271	72 (26.57%)	29	6 (20.69%)	0.493
B	271	89 (32.84%)	29	12 (41.38%)	0.355
AB	271	27 (9.96%)	29	2 (6.90%)	0.841
Rh (+)	253	251 (99.21%)	23	22 (95.65%)	0.231

No. Pts, number of patients; PLC, primary liver cancer; HBV, hepatitis B virus; HCV, hepatitis C virus. Notes to bold values: There were significant differences between the two groups.

**Figure 2:**
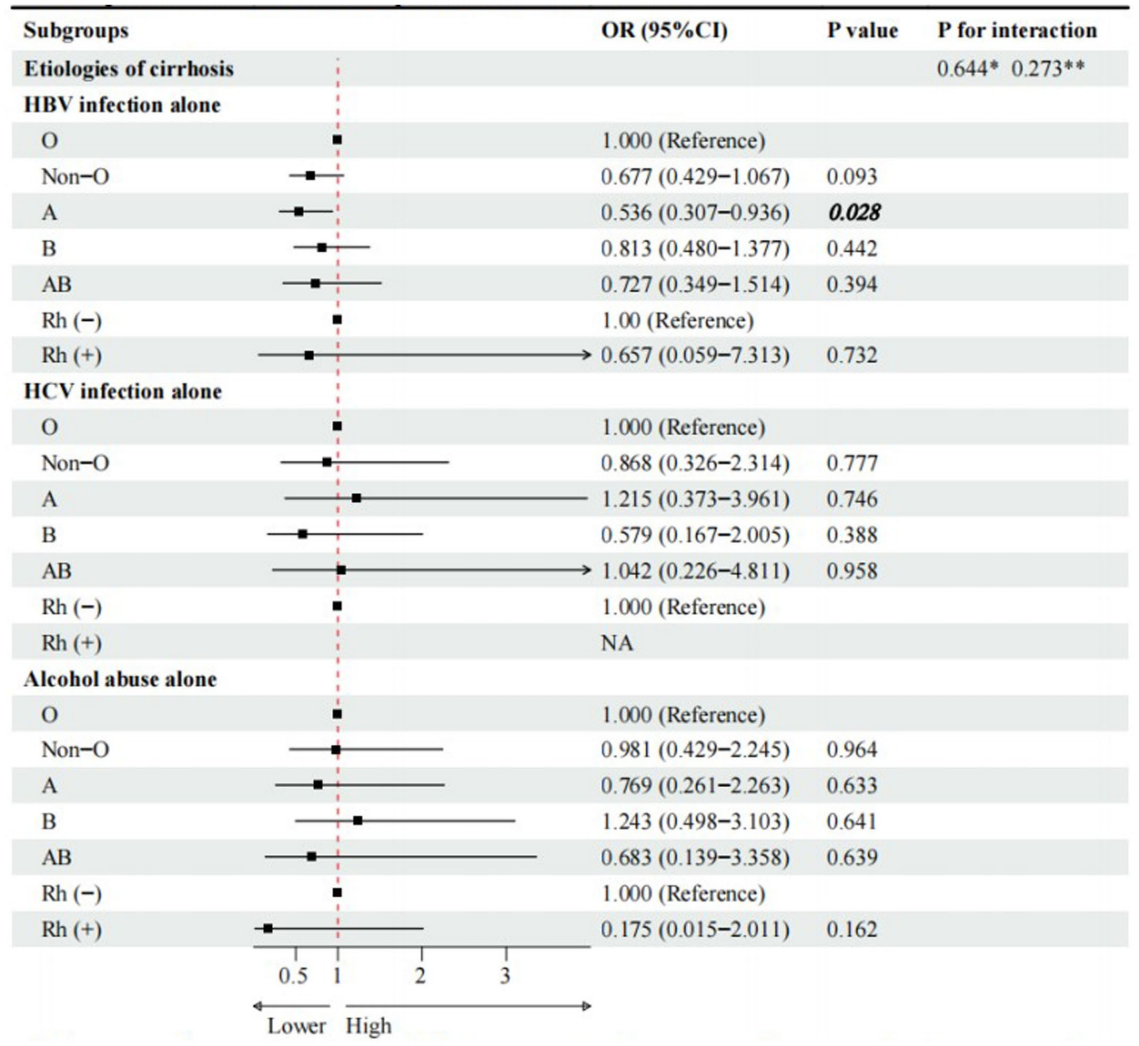
Univariate logistic regression analyses for subgroup analyses of cirrhotic patients with PLC. * Interaction among different etiologies of liver cirrhosis with ABO blood groups. **Interaction among different etiologies of liver cirrhosis with Rh factor. Notes to bold values: There were significant differences between the two groups.

**Figure 3:**
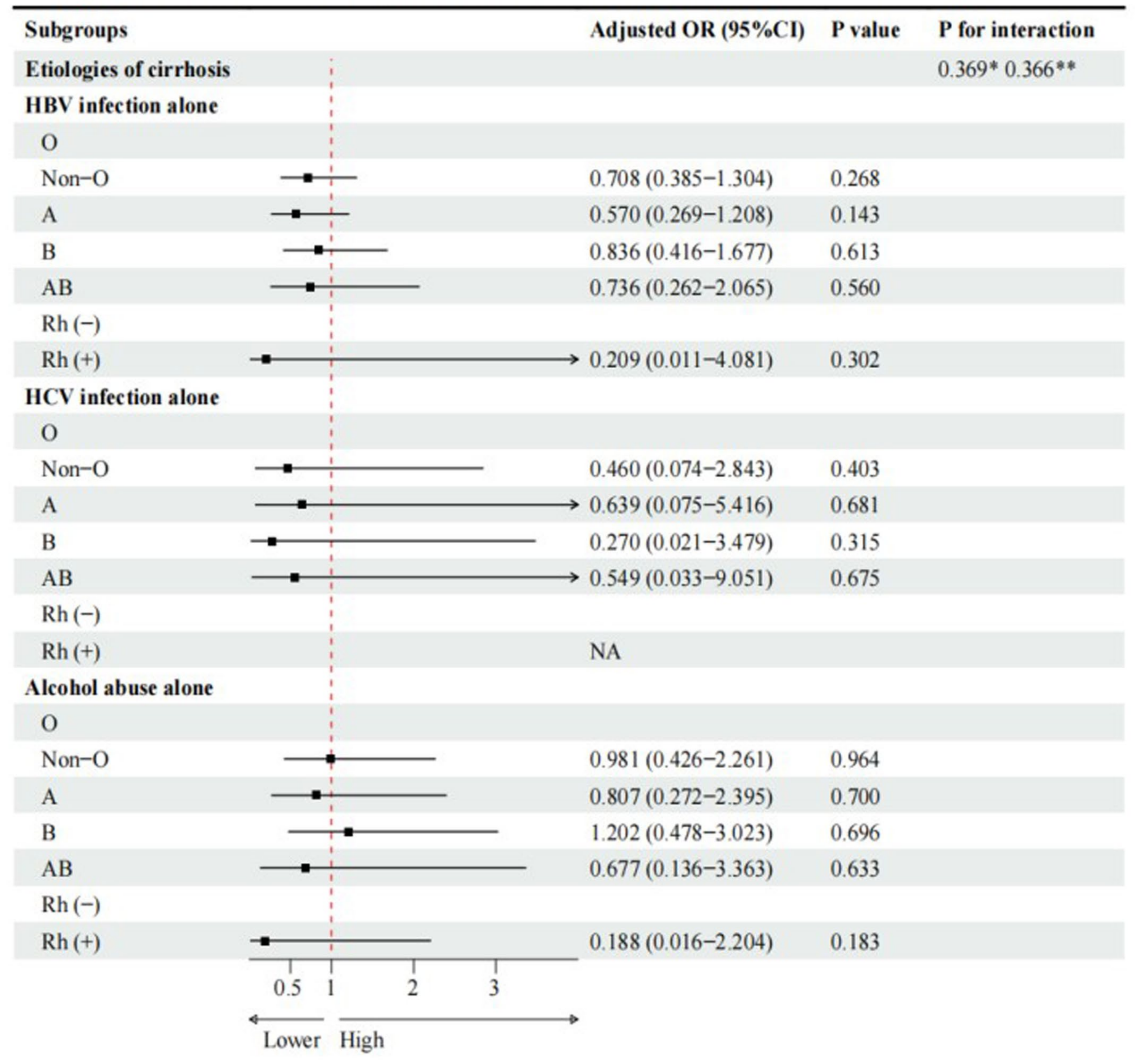
Multivariate logistic regression analyses for subgroup analyses of cirrhotic patients with PLC. Adjusted for gender, age, family history of liver cirrhosis, HBV-DNA. * Interaction among different etiologies of liver cirrhosis with ABO blood groups. **Interaction among different etiologies of liver cirrhosis with Rh factor.

Compared with O blood group, A blood group (cOR = 0.536, 95%CI = 0.307–0.936, *p* = 0.028), rather than non-O, B, and AB blood groups, was significantly associated with a lower risk of PLC in cirrhotic patients with HBV infection alone (Figure 2). However, multivariate logistic regression analyses showed that A blood group was not independently associated with PLC in cirrhotic patients with HBV infection alone (Figure 3).

Compared with Rh (−) blood group, Rh (+) blood group (cOR = 0.657, 95%CI = 0.059–7.313, *p* = 0.732) was not significantly associated with PLC in cirrhotic patients with HBV infection alone (Figure 2). Multivariate logistic regression analysis showed that Rh factor (aOR = 0.209, 95%CI = 0.011–4.081, *p* = 0.302) was not independently associated with PLC in cirrhotic patients with HBV infection alone (Figure 3).

#### 3.4.2 HCV infection alone

In the subgroup of patients with HCV infection alone, the proportions of A, B, O, AB, and Rh (+) blood groups were not significantly different between cirrhotic patients without and with PLC (Table 4).

Compared with O blood group, non-O, A, B, and AB blood groups were not significantly associated with PLC in cirrhotic patients with HCV infection alone (Figure 2). Multivariate logistic regression analyses showed that non-O, A, B, and AB blood groups were not independently associated with PLC in cirrhotic patients with HCV infection alone (Figure 3).

#### 3.4.3 Alcohol abuse alone

In the subgroup of patients with alcohol abuse alone, the proportions of A, B, O, AB, and Rh (+) blood groups were not significantly different between cirrhotic patients without and with PLC (Table 4).

Compared with O blood group, non-O, A, B, and AB blood groups were not significantly associated with PLC in cirrhotic patients with alcohol abuse alone (Figure 2). Multivariate logistic regression analyses showed that non-O, A, B, and AB blood groups were not independently associated with PLC in cirrhotic patients with alcohol abuse alone (Figure 3).

Compared with Rh (−) blood group, Rh (+) blood group (OR = 0.175, 95%CI = 0.015–2.011, *p* = 0.162) was not significantly associated with PLC in cirrhotic patients with alcohol abuse alone (Figure 2). Multivariate logistic regression analysis also showed that Rh factor (aOR = 0.188, 95%CI = 0.016–2.204, *p* = 0.183) was not independently associated with PLC in cirrhotic patients with alcohol abuse alone (Figure 3).

#### 3.4.4 Interaction in subgroup analyses

There was no significant interaction between the etiologies of liver cirrhosis and ABO blood groups or Rh factor (Figures 2, 3).

Collectively, ABO blood groups and Rh factor were not independently associated with PLC in patients with different etiologies of liver cirrhosis.

## 4 Discussion

Our study did not demonstrate any significant association of ABO blood groups and Rh factor with the risk of PLC in cirrhotic patients. Some possible explanations for this finding are as follows: First, the development of PLC is largely influenced by acquired factors but less affected by inherited factors. The majority of our patients had hepatitis B or C virus infection (41.34%) and alcohol abuse (21.46%) as the underlying etiologies of liver diseases, which are also the leading causes of PLC. By comparison, ABO blood groups and Rh factor are inherited characteristics of human populations (14). Second, the development of PLC in cirrhotic patients is largely determined by the regulation of microRNAs, which are a series of small, single-stranded RNAs with approximately 22 nucleotides (15–19) and do not encode the proteins but repress the expression of their target mRNAs on transcriptional and translational levels (20). In cirrhotic patients, multiple signaling pathways and gene expressions can be affected by upgrading the microRNA-21 level and downgrading the microRNA-122, microRNA-29, microRNA-223, and microRNA-193 levels, thereby decreasing cell apoptosis and increasing tumor cell proliferation, invasion, and migration, eventually promoting the development of PLC (21). By comparison, blood groups are differentiated by the presence of A and B antigens on the RBC, which are glycoproteins or glycolipids distributed on the RBC membrane (21) and the products of the gene on chromosome 9q34 (22). Notably, the microRNA level cannot be affected when encoding A and B antigens. Third, the impact of ABO blood groups on PLC may be diluted or even masked by more dominant risk factors, such as hepatitis. Indeed, our previous meta-analysis found that the proportion of O blood group in patients with HCC was significantly lower than that in healthy subjects, but the proportions of ABO blood groups were not significantly different between patients with HCC and hepatitis (23). A case–control study by Shim et al. found that A blood group was associated with a higher risk of PLC in subjects without hepatitis, but ABO blood groups were not significantly associated with PLC in subjects with hepatitis (24). Thus, it can be inferred that ABO blood groups may be associated with PLC in healthy people, but not in patients with hepatitis. Notably, our patients mostly had viral hepatitis, so we did not find any association of ABO blood groups with PLC.

Another finding of our study was that A blood group was a protective factor for PLC in cirrhotic patients with HBV infection alone in univariate logistic regression analyses. However, it should be acknowledged that after adjusting for gender, age, family history of liver cirrhosis, and HBV-DNA, A blood group was not independently associated with PLC in cirrhotic patients with HBV infection alone, which is almost consistent with previous study (11). Therefore, the protective effect of A blood group on PLC, as shown in the univariate analyses, may be caused by confounding factors. Further studies are needed to investigate the association between ABO blood groups and PLC in different settings.

Our study has several advantages as follows. First, to the best of our knowledge, this should be the first study to explore the association of ABO blood groups and Rh factor with the presence of PLC in cirrhotic patients from Liaoning province, China. Second, the selection of eligible patients in our study was more rigorous and reasonable. All subjects included in our study had a diagnosis of cirrhosis. As known, PLC often develops in the setting of cirrhosis. Thus, our data should be more comparable. Third, imaging-based evidence for a definite diagnosis of liver cirrhosis can be obtained in all eligible patients. Thus, our findings should be more accurate and convincing. Fourth, we had a relatively large sample size. Fifth, we adjusted for confounding factors and performed subgroup analyses according to the etiology of liver cirrhosis.

The limitations of our study should not be ignored. First, this was a retrospective cross-sectional study at a single center. Thus, the cause–effect association of ABO blood groups and Rh factor with the development of PLC in cirrhosis could not be clarified. In addition, selection and information bias were inevitable, which may affect the external validity of our study and lead to inaccurate results. Second, most eligible patients included in our study lived in Liaoning province, China. Hence, the findings might be inappropriate to the population from different regions. Third, we did not subdivide the genotypes of the ABO blood groups. In detail, A blood group is composed of AA genotype and AO genotype. B blood group is composed of BB genotype and BO genotype. Therefore, we could not confirm any association of specific ABO genotypes with the risk of PLC in cirrhotic patients.

In conclusion, ABO blood groups and Rh factor may not be associated with the presence of PLC in cirrhotic patients. More large-scale and high-quality prospective studies, multicenter trials, or investigations into the biological mechanisms linking ABO blood groups and Rh factor with PLC are needed to explore the association of ABO blood groups and Rh factor with the risk of PLC among healthy subjects and patients with chronic liver diseases.

## Data Availability

The original contributions presented in the study are included in the article/supplementary material, further inquiries can be directed to the corresponding authors. The datasets presented in this article are not readily available because our data is not open to the public. Requests to access the datasets should be directed to Xingshun Qi, xingshunqi@126.com.
